# Comparing real-world outcomes of total neoadjuvant treatment and CRT at a tertiary medical center

**DOI:** 10.3389/fonc.2023.1305322

**Published:** 2023-11-24

**Authors:** Rim Turfa, Tala Alawabdeh, Ayman Naser, Yazan Alamro, Moath Albliwi, Sama Almasri, Abdullah Al Qazakzeh, Mohammad Abu Shattal, Ali Dabous, Rula Amarin

**Affiliations:** ^1^ Department of Medical Oncology, King Hussein Cancer Center, Amman, Jordan; ^2^ Department of Surgery, King Hussein Cancer Center, Amman, Jordan; ^3^ Department of Internal Medicine, King Hussein Cancer Center, Amman, Jordan; ^4^ Department of Diagnostic Radiology, King Hussein Cancer Center, Amman, Jordan

**Keywords:** total neoadjuvant, complete remission, chemoradiotherapy, locally advanced rectal cancer, recurrence

## Abstract

**Introduction:**

For years, standard treatment for locally advanced rectal cancer (LARC) has included neoadjuvant chemoradiotherapy (CRT), followed by surgery and adjuvant chemotherapy. Although CRT has helped reduce local recurrence rates, it hasn’t consistently improved overall survival. Recent trials have unveiled a different approach called total neoadjuvant treatment (TNT), involving pre-surgery radiotherapy followed by chemotherapy (CAPOX/FOLFOX). TNT shows promise with improved treatment response and lower distant metastasis rates without compromising local control. Consequently, many healthcare institutions have adopted TNT as their preferred neoadjuvant treatment. This study, conducted at a tertiary center, compares the real-world outcomes of both CRT and TNT protocols.

**Methods:**

In this retrospective study of 390 patients treated between 2015 and 2021, aged 18 or older with LARC and tumors within 12 cm of the anal verge, we compared treatment outcomes. We assessed factors like pathological complete remission (pCR), three-year event-free survival (EFS), and overall survival (OS) between the two treatment groups using the Chi-squared test.

**Results:**

Out of the 390 eligible patients, 256 underwent CRT, while 84 received TNT. Surgery was performed on 215 (84%) patients in the CRT group, compared to 55 (65.5%) in the TNT group. Notably, 33 (12.8%) achieved pCR in the CRT group, whereas 23 (27.7%) achieved pCR in the TNT group (P <.001). Regardless of whether surgery was performed or not, the TNT group exhibited lower recurrence rates (12.7% vs. 18.6% with surgery, 28.6% vs. 45% without surgery). The 3-year EFS rate was 80% in the CRT group and 90% in the TNT group (P = .05). Additionally, the 3-year OS rates favored the TNT group, standing at 96.4% compared to 84.4% in the CRT group (P = .005).

**Conclusion:**

Our findings indicate that patients who underwent TNT demonstrated a higher likelihood of achieving pCR and experienced lower recurrence rates compared to those in the CRT group. Additionally, the TNT group exhibited superior 3-year EFS and OS. It is important to note, however, that a longer follow-up period is required to further validate these results.

## Introduction

Managing locally advanced rectal cancer (LARC) presents unique challenges due to the rectum’s anatomical location and the potential surgical complications (1). Over the past decade, numerous trials have explored diverse treatment approaches, including surgery, chemotherapy, and radiotherapy, for patients. However, the optimal sequence of these therapeutic modalities remains an area of ongoing investigation (2).

The Dutch trial led by Kapiteijn and his colleagues, and other similar studies have examined the advantages of incorporating short course radiotherapy into the treatment of locally advanced rectal cancer (LARC) (3). Their research demonstrated that preoperative radiation combined with total mesorectal excision significantly reduces the incidence of local recurrence.

In a 10-year follow-up study of the Dutch trial published in 2012, it was found that the perioperative radiotherapy group experienced more than a 50% reduction in local recurrence compared to the surgery-only group. However, there was no observed improvement in overall survival among the patients (4).

Subsequently, various trials have been conducted to investigate the potential benefits of adding chemotherapy to radiotherapy as a radiosensitizer. These studies have shown promising results in terms of reducing local recurrence rates in patients with locally advanced rectal cancer (LARC) (5).

The German Colorectal Cancer Trial conducted a comparison between preoperative and postoperative chemoradiotherapy (CRT) approaches. The findings revealed significant improvements in the preoperative CRT group in terms of local recurrence rates, sphincter preservation, treatment compliance, and reduced toxicity. However, there was no discernible difference in overall survival between the two groups (6).

As a result, the standard of care for locally advanced rectal cancer (LARC) has long favored preoperative chemoradiotherapy followed by total mesorectal excision (TME) and adjuvant chemotherapy. The Stockholm III trial delved into the impact of delayed versus early surgery on oncological outcomes after short course radiotherapy, uncovering no significant differences in oncologic outcomes but noting fewer postoperative complications in the delayed surgery group (7).

On the other hand, delayed surgery could offer an opportunity for upfront chemotherapy in high-risk patients, particularly those at risk of distant metastasis. Studies have consistently shown that the primary cause of mortality in LARC patients is distant metastasis, with the liver and peritoneum being the most common sites of such metastases (8).

Building on the Stockholm trial’s results, investigators focused on determining the optimal timing for introducing chemotherapy to control micrometastases early in the disease course. This led to the concept of total neoadjuvant therapy (TNT), which combines perioperative chemotherapy and radiotherapy, followed by surgery. TNT was rigorously studied in trials like RAPIDO and PRODIGE (9, 10).

In January 2023, a five-year follow-up update of the RAPIDO trial was published in the Annals of Surgery. It revealed an increased risk of locoregional failure in the TNT arm but a persistent reduction in disease-related treatment failure and distant metastasis.

In this retrospective study, which reflects the experience of a single tertiary cancer center with two distinct treatment modalities for LARC—CRT and TNT—the aim is to report results pertaining to local and distant disease control, pathological complete response, and their effects on event-free survival (EFS) and overall survival (OS) in their patient population.

## Materials and methods

### Study design and participant

This study is retrospective in nature, involving a thorough examination of patient records from those diagnosed with locally advanced rectal cancer within the period spanning from 2015 to 2021. The inclusion criteria encompassed individuals aged 18 years or older who were afflicted with locally advanced rectal cancer (specifically, cT3/4 or T2N+ stage) and who had completed their radiotherapy and chemotherapy protocol.

A total of 390 patient records were meticulously examined, ultimately identifying 340 patients who met the specified inclusion criteria. The study received approval from the relevant institutional review board, and informed consent was appropriately waived.

### Study objectives

The principal objective of this study is to compare the rates of pathological complete response (pCR) between the two study groups. pCR is defined as the absence of viable tumor cells in the pathological assessment. Secondary endpoints include the evaluation of both event-free survival (EFS) and overall survival (OS) over a three-year duration for the two study groups.

### Treatment modalities

In this study patients were staged with CT chest abdomen pelvis, MRI pelvis and sigmoidoscopy to measure the distance from the anal verge.

The CRT regimen comprised neoadjuvant radiotherapy, administered at a dose of 45 Gy divided into 25 fractions over a 5-week period. This was concurrently paired with either capecitabine (825 mg/m2 per dose taken orally twice daily for 7 days a week during radiotherapy) or fluorouracil (225 mg/m2 per day via continuous infusion alongside radiotherapy) over a span of 5 weeks, and 4 months of adjuvant chemotherapy CAPOX/FOLFOX.

Conversely, the TNT regimen encompassed neoadjuvant short-course radiation (5x5 Gy) followed by neoadjuvant chemotherapy, either CAPOX (comprising capecitabine at 1000 mg/m2 orally twice daily on days 1–14 and Oxaliplatin at 130 mg/m2 intravenously every 3 weeks) for 6 cycles. Or nine cycles of FOLFOX4, which included Oxaliplatin at 85 mg/m2 intravenously on day 1, Leucovorin (Folinic acid) at 400 mg/m2 intravenously on day 1, bolus fluorouracil (FU) at 400 mg/m2 intravenously on day 1, and fluorouracil at 1200 mg/m2 intravenously for 48 hours every 2-week cycle.

Following the completion of treatment, patients who achieved clinical complete response (cCR), defined by negative digital rectal examination (DRE), the absence of residual tumor on pelvic MRI, and no detectable viable tumor cells upon biopsy via sigmoidoscopy, were given the choice between a watch-and-wait approach and total mesorectal excision (TME).

### Statistical analysis

The variables were summarized in terms of their median, mean, and range. Cross-tabulation tables were generated to compare and identify distinctions between the two groups in terms of outcomes, including post-surgery recurrence and pathological complete remission.

To illustrate event-free survival (EFS) and overall survival (OS), Kaplan-Meier curves were constructed, and the log-rank test was employed to assess significant differences between the studied groups. Statistical significance was established at a threshold of P<0.05 for all analyses.

EFS was calculated from the commencement of treatment until the occurrence of death or disease recurrence (metastasis or local recurrence). OS was determined by measuring the time from the initiation of treatment until the last follow-up date or the event of death from any cause. All statistical analyses were conducted using the SPSS software.

## Results

### Patient characteristics

In this retrospective study, a cohort of 340 patients met the eligibility criteria, comprising 256 individuals in the CRT group and 84 individuals in the TNT group. The median age for the entire patient population was 56 years for the CRT group and 57 years for the TNT group. The two groups exhibited comparability across all characteristics. The median duration of follow-up was 36 months for the TNT group and 72 months for the CRT group. A summary of patient characteristics can be found in **Table 1**.

**Table 1:** Patient demographics.

Variables	Treatment Modality	
	CRT	TNT
Median age, years (range)	56 (20-82)	57 (31-83)
Sex, n (%)		
Male	148 (58)	52 (62)
Female	108 (42)	32 (38)
clinical stage, n (%)		
T2N1	11 (4)	4 (5)
T2N2	6 (2)	5 (6)
T3N0	4 (2)	2 (2)
T3N1	44 (18)	3 (4)
T3N2	143 (58)	54 (66)
T4N1	1 (0.4)	0 (0.0)
T4N2	38 (15)	14 (17)
Distance from anal verge		
0-5 cm	106 (41)	37 (44)
5.1-10 cm	130 (51)	38 (45)
> 10 cm	20 (8)	9 (11)

CRT, Chemoradiation therapy; TNT, total neoadjuvant therapy.

### Pathologic and survival outcomes

Out of the 256 patients in the CRT group, 33 (12.8%) achieved pathological complete remission (pCR), which was notably lower than the TNT group where 23 out of 84 patients (27.7%) achieved pCR (P <.001). In the TNT group, 17 patients (20.5%) reached clinical complete remission (cCR) and were subsequently managed through a “watch and wait” approach, while in the CRT group, 17 patients (6.6%) achieved cCR (P <.001).

Among the patients in the CRT group who underwent surgery (n=215), 40 individuals (18.6%) encountered disease recurrence, in contrast to 19 patients (45%) who did not undergo surgery (P <.001). In the TNT group, out of the 55 patients who underwent surgery, seven cases (12.7%) experienced disease recurrence following the surgical procedure. Conversely, among the eight patients who did not undergo surgery in the TNT group, 28.6% experienced disease recurrence, although this difference did not reach statistical significance (P = .07). Further details can be found in **Table 2**, illustrating these outcomes.

**Table 2:** Treatment outcome.

Variables	Treatment Modality	
	CRT (%)	TNT (%)
**Pathologic complete remission, n (%)**	33(12.8)	23(27.7) (P < 0.001)
**Clinical complete response,**	17(6.6)	17(20.5) P <0.001
**Residual disease, n (%)**	203 (79)	45(54.2)
Surgery, n (%)		
**Yes**	215 (83.7)	55 (66)
**No**	42 (16)	28(33.7)
Reasons no surgery		
**Died before finishing chemoradiotherapy**	1 (2.4)	0 (0)
**Disease progression**	11 (26)	5 (19)
**Refused surgery**	13 (31)	4 (15.4)
**Watchfull waiting**	17 (40.5)	17 (65.4)
**Recurrence in general**	59 (23)	15 (18) P 0.3
**Local**	24(40.7)	4(26.7) 0.59
**Distant**	35(59.3)	11(73.3) P 0.15
**Recurrence after surgery, n (%)**	40 (18.6)	7 (12.7)
**Recurrence without surgery, n (%)**	19 (45) P<0.001	8 (28.6) P 0.07
Recurrence according to distance from anal verge		
**0-5 cm**	28(26)	5 (13.9) P 0.13
**5.1-10 cm**	26(20)	9 (23.7) P 0.6
**> 10 cm**	5(25)	1 (11) P 0.39
Stoma		
**Yes**	82(31.9)	18(21.7)
**No**	175(68.1)	65(78.3)
		P 0.076

CRT, Chemoradiation therapy; TNT, total neoadjuvant therapy.

It’s worth noting that the TNT group exhibited a higher rate of distant metastasis (73.3%) compared to the CRT group (59.3%), although this difference did not reach statistical significance (P = .15). Conversely, the local recurrence rate was lower in the TNT group (26.7%) two of them were on watch and wait group and were managed surgically, compared to the CRT group (40.7%), but this difference also lacked statistical significance (P = .59).

When considering tumor location, tumors located more than 10 cm from the anal verge demonstrated a lower recurrence rate in both treatment groups, although this difference was not statistically significant (P = .39), as illustrated in **Table 2**.

Regarding the necessity for a permanent stoma, 82 patients (31.9%) in the CCRT group ultimately required a permanent stoma, in contrast to 18 patients (21.7%) in the TNT group. While this trend suggested a difference, the statistical significance remained borderline (P = .07), as detailed in **Table 2**.

**Figure 1:**
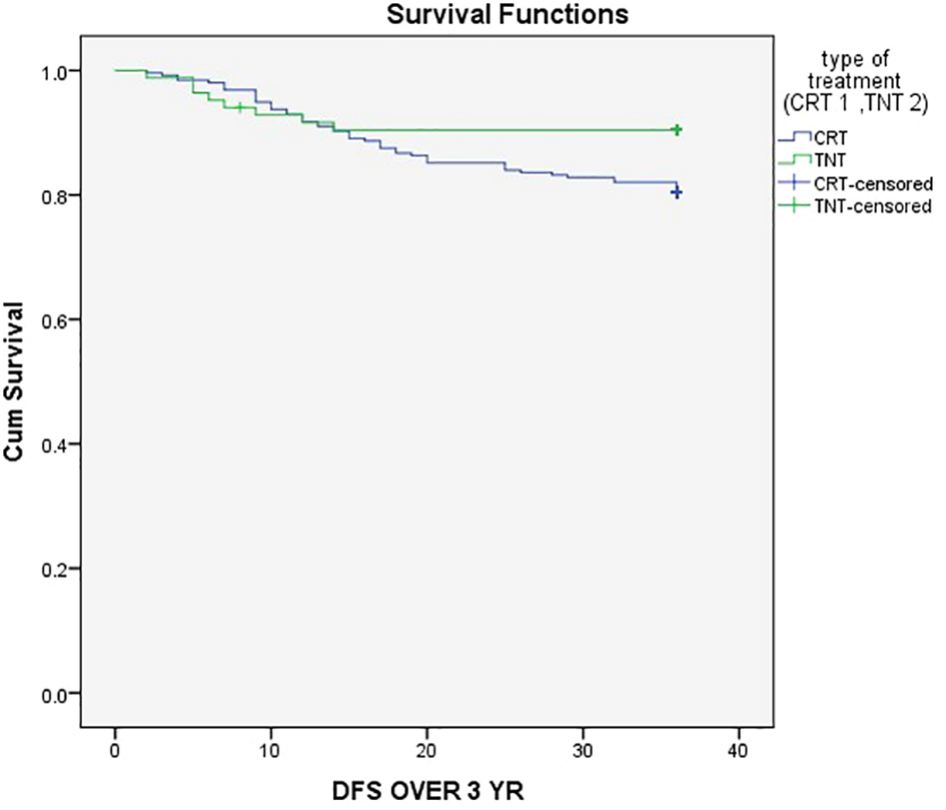
Three-year event-Free Survival (EFS) rate was 80% in the CRT group compared to 90% in the TNT group.

**Figure 2:**
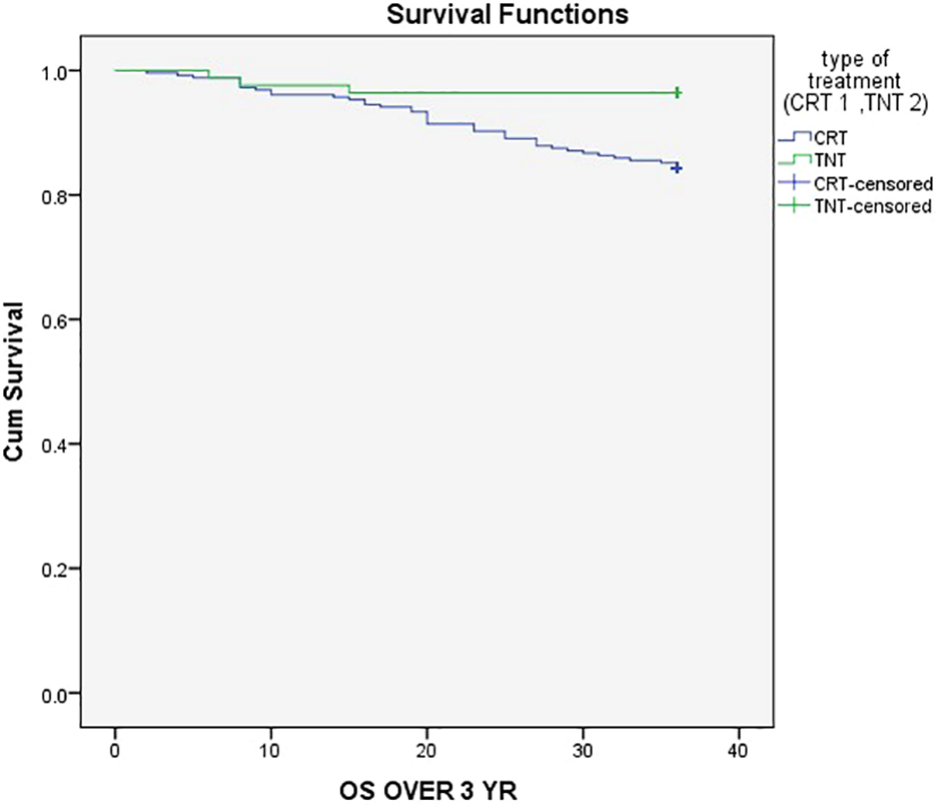
Three-year Overall Survival (OS) rates were 84.4% in the CRT group and 96.4% in the TNT group.

The three-year event-Free Survival (EFS) rate was 80% in the CRT group compared to 90% in the TNT group (P = .05), as demonstrated in **Figure 1**. Similarly, the three-year Overall Survival (OS) rates were 84.4% in the CRT group and 96.4% in the TNT group (P = .005), as depicted in **Figure 2**.

## Discussion

The primary objectives in the management of patients with locally advanced rectal cancer (LARC) revolve around reducing both local and distant treatment failures while striving to enhance event-free survival (EFS) and extend overall survival (OS). Despite efforts to introduce adjuvant chemotherapy after surgery, there has been limited success in significantly improving EFS and OS, even with the inclusion of oxaliplatin alongside capecitabine (11).

In our dataset, we observed a higher pCR rate among patients who underwent surgery in the TNT group compared to the CRT group (27.7% vs. 12.8%) with a highly significant difference (P <.001). Furthermore, there was a trend toward lower recurrence after a 3-year follow-up period in the TNT group compared to the CRT group (18% vs. 23%), although statistical significance was not reached (P = .3). This trend was also observed when comparing patients with high rectal tumors (>10cm) to those with tumors <10cm in both treatment groups, as detailed in **Table 2**. Additionally, the clinical complete response (cCR) was notably higher in the TNT group compared to the CRT group (20.5% vs. 6.6%), with a highly significant difference (P <.001).

Also, we observed a slightly higher rate of distant metastasis and a lower rate of local recurrence in the TNT group compared to the CRT group, although these differences did not achieve statistical significance. Interestingly, these findings diverge from the updated data reported in the RAPIDO trial, published in January 2023 in the Annals of Surgery.

Conversely, findings from the German CAO/ARO/AIO-04 phase III trial have demonstrated that the incorporation of oxaliplatin into preoperative chemoradiation followed by adjuvant fluorouracil leads to a noteworthy enhancement in DFS and OS (12).

With regard to the incidence of local recurrence, a study conducted by Ngan et al. revealed that there were no notable disparities in the 3-year local recurrence rates, overall survival (OS), distant recurrence rates, or late toxicity when comparing short-course radiotherapy (25 Gy delivered in 5 fractions) to long-course chemoradiation (50.4 Gy administered in 28 fractions) (13). Moreover, findings from the Polish II randomized trial indicated that there were no discernible distinctions in terms of OS or disease-free survival (DFS) between patients who underwent short-course radiotherapy followed by neoadjuvant chemotherapy and those who received upfront chemoradiotherapy (14).

Total neoadjuvant treatment was further evaluated by the famous RAPIDO trial that compared short-course radiotherapy followed by neoadjuvant chemotherapy and subsequent total mesorectal excision (TME) against long-course radiotherapy followed by TME, with optional adjuvant chemotherapy. The RAPIDO trial yielded noteworthy results, including a significantly superior pathological complete response (pCR) rate (28% vs. 14%) and a reduction of 7% in disease-related treatment failures, favoring the total neoadjuvant therapy (TNT) arm. Interestingly, the 3-year overall survival (OS) rate remained consistent across both groups (9).

A systematic review further supported these findings, reporting pooled pCR rates of 32.4% for TNT and 22.3% for CRT, underscoring the benefits of the total neoadjuvant therapy approach (15).

Moreover, in our patients the requirement for a permanent stoma was notably lower in the TNT group compared to the CRT group (21.7% vs. 31.7%), although this difference approached but did not reach statistical significance (P = .076). This observation implies potential benefits in terms of reduced morbidity and improved quality of life for patients in the TNT group (16).

In our study, there was a notable and statistically significant difference in the 3-year event-free survival (EFS) between the two treatment groups, with rates of 90% in the TNT group and 80% in the CRT group (**Figure 1**) (P = .05). This finding ligns with the results of a randomized trial that demonstrated improved EFS in the TNT group (17).

Additionally, our data revealed a significant difference in 3-year overall survival (OS) between the TNT and CRT groups, with rates of 84.4% and 96.4%, respectively (**Figure 2**) (P = .005). This outcome was consistent with the findings from the randomized STELLAR trial, which also reported better 3-year OS in the TNT group compared to CRT (86.5% vs. 75.1%; P = .033) (18).

However, it’s worth noting that Goffredo et al. reported no superiority of TNT over CRT in their extensive cohort comprising more than 8000 patients (19).

In recent times, immune checkpoint inhibitors have garnered significant attention in the field of rectal cancer treatment. Cercek et al. conducted a study on neoadjuvant Dostarlimab in patients with MMR-D locally advanced rectal cancer, revealing an impressive 100% clinical complete response (cCR) rate after 12 months of follow-up (20). Additionally, a phase II trial explored the combination of Avelumab (anti-programmed death-ligand 1) with neoadjuvant mFOLFOX following short-course radiotherapy before surgery, yielding promising results with a notable pathological complete response (pCR) rate and a major response rate (21).

One limitation of our study stems from its retrospective nature, which relied on chart review, and the relatively small sample size, particularly in the TNT arm, and the missing data of MSI. Therefore, a larger patient cohort with extended follow-up duration is essential to validate our findings regarding recurrence rates, event-free survival (EFS), and overall survival (OS).

In conclusion, our data indicate that total neoadjuvant therapy (TNT) surpasses conventional chemoradiotherapy (CRT) in terms of achieving pathological complete response (pCR), reducing local tumor recurrence, preserving organs, and enhancing 3-year EFS and OS. Nevertheless, to reconcile the conflicting data concerning disease-free and OS rates between these two treatment modalities, further studies are warranted.

## Data availability statement

The data analyzed in this study is subject to the following licenses/restrictions: It’s owned by KHCC and includes personal information and we are not allowed to share. Requests to access these datasets should be directed to IRBOFFICE@khcc.jo.

## Ethics statement

The studies involving humans were approved by King Hussein Cancer Center institutional review board. The studies were conducted in accordance with the local legislation and institutional requirements. Written informed consent for participation was not required from the participants or the participants’ legal guardians/next of kin in accordance with the national legislation and institutional requirements.

## Author contributions

RT: Writing – review & editing, Writing – original draft. TA: Data curation, Writing – original draft, Writing – review & editing. AN: Writing – review & editing. YA: Data curation, Writing – review & editing. MA: Data curation, Writing – review & editing. SA: Data curation, Writing – review & editing. AA: Data curation, Writing – review & editing. MS: Writing – review & editing. AD: Writing – review & editing. RA: Writing – review & editing.
